# Supplementary material for: Aeration strategy at birth does not impact carotid haemodynamics in preterm lambs
Source: Pediatr Res. 2022 Aug 16;93(5):1226–32. doi: 10.1038/s41390-022-02244-z (PMC10132978; doi:10.1038/s41390-022-02244-z)
Supplement: Supplementary file 2 — Dahm_CBF_Online supplementary data_R1_20Jul22 [file 41390_2022_2244_MOESM2_ESM.pdf]

Aeration Strategy at Birth Does Not Impact Carotid Blood Flow and Oxygen Delivery in Preterm Lambs

Sophia I Dahm[1*], Kelly R Kenna[1], David Stewart[1,2], Prudence M Pereira-Fantini[1,3], Karen E McCall[1,2], Liz Perkins[1,2], Magdy Sourial[1], David G Tingay[1,2,3]

Online Data Supplement

Online Table 1. Dynamic 14 cmH_2O and 20 cmH_2O PEEP Group Lamb Characteristics

Online Figure 1. Absolute carotid blood flow (CBF) waveform measures for the 14 cmH_2O and 20 cmH_2O maximum PEEP Dynamic strategies.

Online Figure 2. Absolute carotid artery oxygen (CAO) content and carotid oxygen delivery for the 14 cmH_2O or 20 cmH_2O maximum PEEP Dynamic strategies.

Online Table 1. Dynamic 14 cmH_2O and 20 cmH_2O PEEP Group Lamb Characteristics

Dynamic PEEP Group	n	GA (d)	Weight (kg)	Gender (F:M)	Parity (S:T)	Fetal Fluid (ml/kg)	Static C_{RS} (ml/kg/cmH_2O)	Fetal Arterial Blood Gas				Cerebral Blood Flow (ml/kg/min)			
								pH	$PaCO_2$ (mmHg)	PaO_2 (mmHg)	BiC (mmol)	Height	Min	Mean	Max
**Low**	14	125.5 (1.0)	3.52 (0.32)	8:6	0:14	16.6 (5.0)	1.18 (0.22)	7.36 (0.07)	47.0 (7.4)	27.9 (3.0)	24.2 (2.7)	52.3 (14.2)	-0.6 (5.1)	15.0 (5.7)	50.7 (14.9)
**High**	27	125.2 (1.0)	3.33 (0.32)	18:9	3:24	17.8 (5.2)	1.22 (0.19)	7.34 (0.06)	47.5 (6.9)	27.3 (6.3)	23.0 (2.6)	50.3 (17.9)	-0.1 (3.8)	14.0 (6.1)	50.3 (16.6)

Abbreviations: GA; gestational age, F; female, M; male, S; singleton, T; multiparity, C_{RS}; compliance, $PaCO_2$; partial arterial pressure of carbon dioxide, PaO_2; partial arterial pressure of oxygen, Bic; bicarbonate. All data mean (SD) or ratio. All p values one-way ANOVA or chi-test as appropriate.

Online Figure 1. Absolute carotid blood flow (CBF) waveform measures for the 14 cmH_2O and 20 cmH_2O maximum PEEP Dynamic strategies.

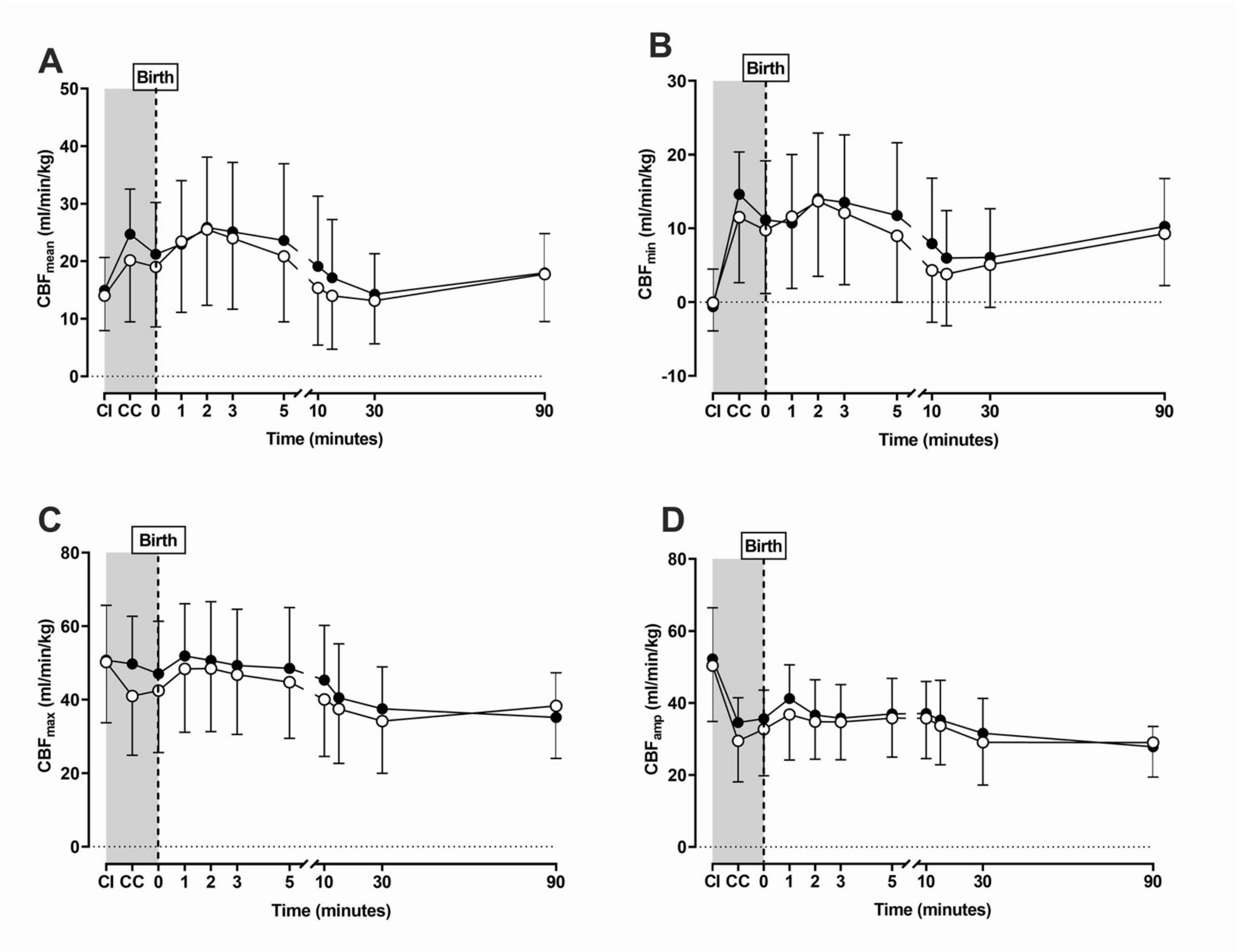

Online Figure 1: Absolute carotid blood flow (CBF) waveform measures including the mean (**A**; p=0.9479), minimum (**B**; p=0.9043), maximum (**C**; p=0.6684) and amplitude (**D**; p=0.9024) in the 14 cmH_2O (black circles) or 20 cmH_2O (white circles) maximum PEEP Dynamic strategies. All p values overall mixed effects model (time and strategy combined). There was no difference in the groups at each time point (all p>0.05; Tukey post-tests). Fetal period without any ventilation shown in grey background. CI; cord intact, CC; post-cord clamping, Birth; first 10s after commencing allocated recruitment strategy. All data mean and standard deviation.

Online Figure 2. Absolute carotid blood flow, carotid artery oxygen (CAO) content and carotid oxygen delivery for the 14 cmH_2O or 20 cmH_2O maximum PEEP Dynamic strategies.

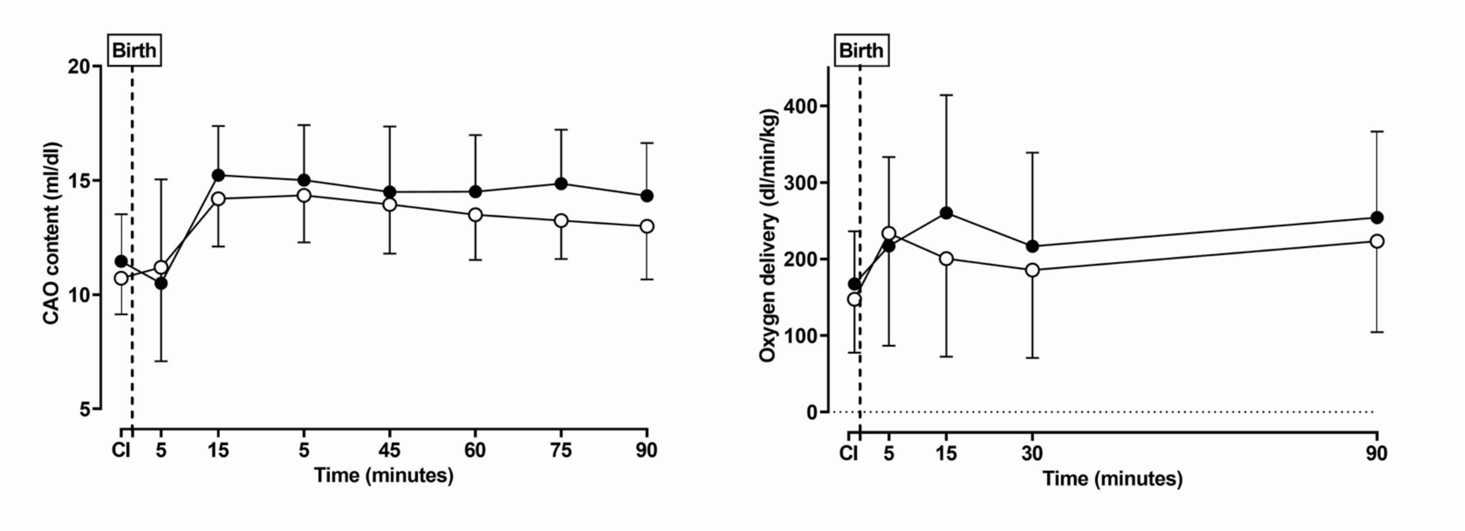

Online Figure 2: Absolute carotid arterial oxygen (CAO) content (**A;** p=0.2712) and carotid oxygen delivery (**B;** p=0.2800) in the 14 cmH_2O (black circles) or 20 cmH_2O (white circles) maximum PEEP Dynamic strategies. P values calculated with an overall mixed effects model (time and strategy combined). There was no difference in the groups at each time point (all $p>0.05$; Tukey post-tests). CI; cord intact, CC; post-cord clamping, Birth; first 10s after commencing allocated recruitment strategy. All data mean and standard deviation.
